# HIV, Antiretroviral Therapy and Metabolic Alterations: A Review

**DOI:** 10.7759/cureus.8059

**Published:** 2020-05-11

**Authors:** Huseyin Ekin Ergin, Evelyn E Inga, Tun Zan Maung, Mehwish Javed, Safeera Khan

**Affiliations:** 1 Medicine, California Institute of Behavioral Neurosciences and Psychology, Fairfield, USA; 2 Internal Medicine, California Institute of Behavioral Neurosciences and Psychology, Fairfield, USA; 3 Internal Medicine, LaSante Health Center, Brooklyn, USA

**Keywords:** antiretroviral, hiv, metabolic syndrome, hypertension, insulin resistance, dyslipidemia

## Abstract

The introduction of antiretroviral therapy (ART) has caused some metabolic problems to people who suffer from HIV. ART probably is not the sole reason for these metabolic disorders. Most likely, HIV itself affects the metabolism as well. We conducted research to find the prevalence of the different types of metabolic disorders among HIV(+) patients. Female gender, high BMI, and older age are among the risk factors for the occurrence of metabolic disorders. Regarding dyslipidemia, hypertriglyceridemia and low high-density lipoproteins (HDLs) are the most common types of dyslipidemia in the studies we included. Protease inhibitors (PIs) are widely known as the most common class of antiretroviral drugs that cause metabolic disorders, and some studies in our review also demonstrated this knowledge. In our review, we concluded that HIV and ART concurrently alter the metabolism, but further research is required about this substantial topic.

## Introduction and background

On the report of the Joint United Nations Programme on HIV/AIDS (UNAIDS), there were approximately 37.9 million people who were HIV(+) at the end of 2018 [1]. Also, nearly 24.7 million people were able to access antiretroviral therapy [1]. The emergence of antiretroviral therapy (ART) in 1996 has increased the life expectancy and life quality of people who have HIV, and the HIV disease is considered a chronic condition [2]. According to the World Health Organization (WHO)’s updated guidelines in 2018, first-line ART for adults usually consists of two nucleoside/nucleotide reverse transcriptase inhibitors (NRTIs) + integrase inhibitors [3]. The second-line ART for adults is usually consisting of two NRTIs + protease inhibitors (PIs) [3].

Adult Treatment Panel III (ATP III) report identified metabolic syndrome (MS) as a group of metabolic abnormalities, which includes: abdominal obesity, increased triglycerides, high blood pressure, increased fasting glucose, and decreased high-density lipoproteins (HDLs) [4]. If three or more criteria are met, MS is present [4]. According to Moore et al., the prevalence of MS among the United States adults has increased by more than 35% from 1988-1994 to 2007-2012, rising from 25.3% to 34.2% [5]. It is also known that MS increases cardiovascular disease (CVD) risk, and it is associated with insulin resistance, prothrombotic state, and proinflammatory state [4].

Recent studies suggested that HIV increases the risk of metabolic disorders even before the introduction of ART [6-11]. Some studies also demonstrated that ART-initiation increases the risk while HIV was not a big risk factor itself [12-18]; another group of studies proposed that HIV and ART increase the risk together [19-21]. At the other end of the spectrum, some studies propose combined ART may have good effects on HIV(+) patients who also meet the criteria for MS [22]. Some studies also report that newer antiretroviral drugs decrease the prevalence of insulin resistance among HIV(+) patients compared to old therapies [23]. PIs are generally well-known for these side effects [15,20,24-26]; also, some NRTIs, especially stavudine, may cause long-term metabolic and cardiovascular complications [24]. Another study did not find PIs as an important contributor to CVD [12]. Some studies compared two NNRTIs for their metabolic effects [27]. A study from Korea has not found a correlation between the use of NNRTI and dyslipidemia [28]. There is also some evidence about the hepatitis C virus (HCV), boosting the risk of these abnormalities caused by HIV/ART [29]. Older age [6,12,14,15,19,20,30,31], high BMI [6,14,16,28] and female gender [6,9,15,19,20,30] are also among the factors, which independently increases the risk of MS in patients with HIV/on ART. Table 1 contains the baseline BMI values of ART-exposed and ART-naive patients in some of the articles.

**Table 1:** Mean baseline BMI of the ART-exposed and ART-naive patients ART, antiretroviral therapy

Author	Treatment naive	Treatment exposed
Osoti et al. (2018) [6]	23.8 ± 5.2 kg/m^2^ (mean ± SD)	25.1 ± 5.9 kg/m^2 ^(mean ± SD)
Muhammad et al. (2017) [14]	21.6 ± 5.6 kg/m^2^ (mean ± SD)	21.3 ± 4.4 kg/m^2 ^(mean ± SD)
Calza et al. (2017) [16]	23.8 ± 5.9 kg/m^2^ (mean ± SD)	24.4 ± 5.6 kg/m^2^ (mean ± SD)
Oh et al. (2017) [28]	14.8 – 33.8 kg/m^2 ^(range)	14.5 – 37.8 kg/m^2^ (range)

ART can also cause lipodystrophy syndrome [32]. It is defined by changes in adipose tissue and redistribution of fat from the periphery (face, buttocks, legs, arms) to the abdomen, neck, and breasts. Some studies have found that lipodystrophy is more common with dyslipidemia [29,32-34], while some have not found a meaningful relation between dyslipidemia and lipodystrophy [35].

Our study group has reached a consensus about the lack of studies that compare the frequency of HIV/ART-related metabolic alterations and its consequence, CVD. Also, there are not enough studies that explain the pathophysiology of metabolic alterations among HIV(+) patients on ART.

In this review article, we tried to sum up the recent information about the mechanisms, epidemiology, and metabolic effects of different regimens of ART and other factors affecting HIV/ART-related MS and its consequence, CVD.

## Review

Methods

We conducted a comprehensive literature review via PubMed, PubMedCentral, Google Scholar, and ResearchGate. We used keywords antiretroviral, HIV, MS, hypertension, insulin resistance, dyslipidemia, both alone and in combination to look for the research papers. Our entire database included studies that only focused on the human population. Studies that were other than the English language were excluded. We have included all the full-text articles except one, in our review. Our whole data were collected ethically and legally. 

Discussion

HIV infection is one of the most common diseases in the world; in 2018, there were about 37.9 million people who suffered from this condition, of whom 24.7 million were able to access ART according to UNAIDS [1]. ART has been a great improvement for the survival of these patients [2], but this therapy is also associated with some adverse metabolic effects as well [12-14]. HIV itself may be related to metabolic effects as well [6-8]. Besides, we already know that metabolic disorders can increase the risk of CVD, which is the most common cause of mortality worldwide, according to WHO [4,36].

**Figure 1:**
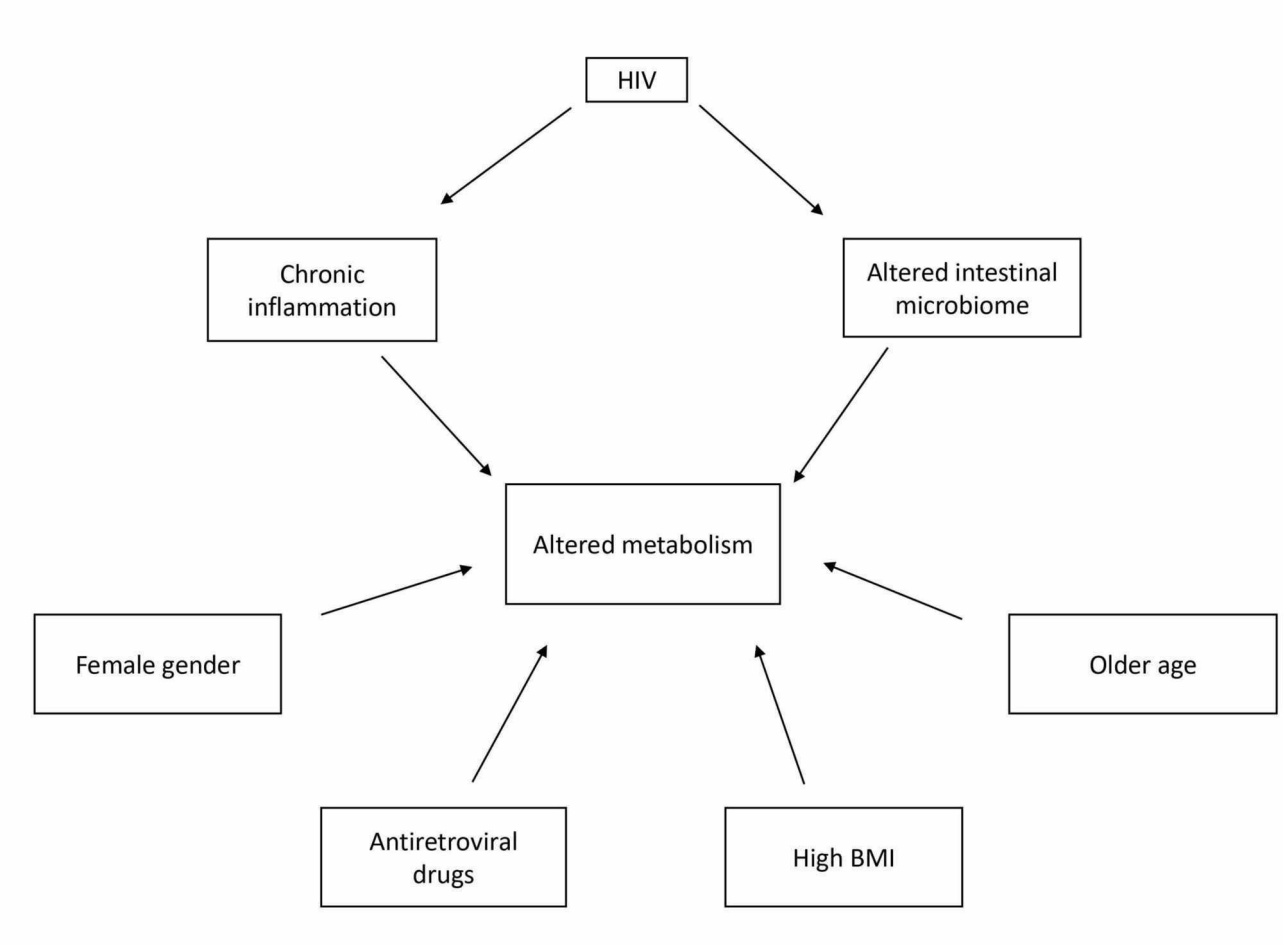
Factors associated with metabolic alterations in HIV(+) patients

The way HIV and ART alter the metabolism is not completely clear; these disorders are possibly from the coalescence of inflammation caused by the virus, altered intestinal flora, ART, and traditional risk factors such as old age (Figure 1). Monaco et al. suggested that in patients with untreated HIV infection and low CD4 T-lymphocyte levels, the intestinal microbiome may be altered [37]. Dillon et al. and Brenchley et al. stated that the altered microbiome is associated with systemic inflammation; these findings may be the reason for altered metabolism [38,39]. Peltenburg et al. have found impaired biogenic amine levels in an untreated HIV(+) patient group [40]. Metabolic dysregulation was also common in this study; biogenic amine disturbances may have a role in the metabolic effects of HIV as well. According to Duro et al., HIV patients with MS had increased levels of C-reactive protein and interleukin-6 [21]. HIV-related metabolic disturbances were more prevalent in female gender in our studies [6,9,15,19,20,30], old age was also an independent risk factor in some of the studies [6,12,14,15,19,20,30,31], and according to some studies, high BMI was a risk factor by itself [6,12,14,16,28].

The pathogenesis of HIV/ART-related metabolic disturbances is not fully known. As we indicated, there are theories on this subject, such as the chronic inflammation of HIV or alterations in gut flora, but these theories are not proven. Nonsteroidal anti-inflammatory drugs (NSAIDs) can be used to reduce chronic inflammation and may reduce metabolic alterations. Clinical trials using NSAIDs can be conducted to see the effect of chronic inflammation on metabolism better. Antibiotics may affect the gut flora, and clinical trials about the effect of antibiotics and gut flora dysbiosis on the subject can be performed as well.

Dysglycemia

According to WHO, there were about 422 million people who were suffering from diabetes mellitus (DM); there are more people who suffer from impaired plasma glucose when we add the prediabetic population into the equation [41]. Insulin resistance prevents insulin-dependent glucose entry. As per American Diabetes Association’s new guidelines in 2020, criteria defining DM are fasting plasma glucose (FPG) level of >126 mg/dL after minimum eight hours of fasting, plasma glucose (PG) >200 mg/dL two hours after 75-gram oral glucose tolerance test (OGTT) and hemoglobin A1c (HbA1c) level of >6.5%; prediabetes criteria are FPG between 100 and 125 mg/dL, PG two hours after OGTT between 140 and 199 mg/dL and HbA1c level of 5.7% to 6.4% [42]. There is quite a bit of possibility that HIV or ART may affect the blood glucose levels and insulin resistance; most of the studies we collected have demonstrated this information. Table 2 contains the studies we collected for dysglycemia.

**Table 2:** Selected studies about dysglycemia in the review NRTI, nucleotide reverse transcriptase inhibitor; NNRTI, non-nucleoside reverse transcriptase inhibitor; PI, protease inhibitor; ART, antiretroviral therapy; DM, diabetes mellitus; MS, metabolic syndrome; HDL, high-density lipoprotein; HAART, highly active antiretroviral therapy; CVD, cardiovascular disease; HCV, hepatitis C virus

Author	Drug studied	Number of patients	Type of study	Result	Conclusion
Bune et al. (2019) [31]	NRTI + NNRTI + PI	633	Cross-sectional study	31.3% of the ART-exposed patients had DM. DM was prevalent in %28 of the ART-naive patients. DM was the third most frequent component of the MS.	MS was more frequent in ART-exposed patients than ART-naive patients in this study.
Padmapriyadarsini et al. (2018) [7]	-	390	Cross-sectional study	Increased rate of insulin resistance (17%) was identified with ART-naive children.	Prevalence of cardiometabolic disorders was higher in this group, children should be monitored for metabolic disorders after the initiation of ART.
Raposo et al. (2017) [8]	-	87	Cross-sectional study	The most common metabolic disorders in the patient group were low HDL cholesterol, hypertriglyceridemia and abdominal obesity. A major portion of the patients was in the low-risk group regarding the CVD risk.	Metabolic disorders were relatively high in this patient group, initiation of ART may increase the prevalence even higher.
Muhammad et al. (2017) [14]	ART	300	Cross-sectional study	MS was more prevalent in patients on HAART than HAART-naive patients. Duration of HAART exposure wasn’t significantly associated with insulin resistance.	HAART, especially regimens with PIs was associated with the increased risk of MS.
Kingery et al. (2016) [13]	The first-line ART regimen was either tenofivir/emcitrabine or zidovudine/lamivudine+nevirapine or efavirenz. Protease inhibitors were used as second-line ART.	454	Cross-sectional study	MS was more prevalent in HIV(+) patients on ART. High fasting blood glucose was more common in patients on ART.	MS develops more frequently in the first 3-4 years of ART. CVD risk is also increased concurrently with MS.
Levitt et al. (2016) [20]	First-line ART (stavudine (d4T), lamivudine (3TC), and efavirenz or nevirapine), and second-line ART (zidovudine (AZT), didanosine (ddI) and lopinavir– ritonavir (LPV-r)).	1820	Cross-sectional study	Dysglycemia prevalence was 37.0% of patients on second-line ART, 26.0% on first-line ART, 21.6% on ART-naive and 18.0% on community-based survey. High dysglycemia risk was related to older age and HIV status. Patients on second-line ART were in the highest risk category for dysglycemia.	HIV and ART were found to be a risk factor for dysglycemia in this study. Routine screening should be done for HIV(+) patients.
Maganga et al. (2015) [18]	ART	454	Cross-sectional study	Impaired blood glucose was more common in ART-exposed patients than HIV(-) group. Percentage of glucose metabolism disorders was similar in ART-naive and HIV(-) but this finding was not statistically significant.	Risk of glucose metabolism disorders was four times higher in ART-exposed patients compared to HIV(-) controls.
Mbunkah et al. (2014) [12]	First-line drug treatment was a combination of two NRTIs + an NNRTI, while second-line drug treatment was a combination of two NRTIs + 2 PIs.	223	Cross-sectional study	Hyperglycemia and MS were more common in patients on ART than ART-naive patients and uninfected individuals. Prevalence of MS was higher in females than in males. Patients on first-line drugs had a higher ratio of MS than patients on second-line therapy. Lamivudine/stavudine/nevirapine were the highest risk of drugs for MS.	ART but not HIV increases the risk of MS.
Araujo et al. (2014) [23]	Predominance of PI in pretreated patients (14 vs 56%), while most first-line patients received non-nucleoside analogs (86 vs 41%). Specifically, DRV or ATV was primarily used in pretreated patients	265	Cross-sectional study	Insulin resistance was found to be less prevalent in patients on first-line treatment compared to pretreated patients.	Newer antiretrovirals were demonstrated to be safer than older drugs considering metabolic disorders.
Jain et al. (2007) [29]	ART	1529	Observational	Race, age, high BMI and HCV were risk factors for DM in HIV(+) patients. PIs were not demonstrated as a risk factor for DM.	HCV is shown to be a distinct risk factor for DM in this patient group.

Several studies suggested that HIV increases the risk of impaired glucose metabolism, even without the use of ART. Padmapriyadarsini et al. conducted a cross-sectional study of ART-naive children in India to assess the associations between HIV and metabolic effects. In their study group, the prevalence of insulin resistance was high, 17%. According to this study, low height-for-age Z-score and weight-for-age Z-score were significantly associated with the development of insulin resistance (p = 0.01 and p = 0, respectively) [7].

Raposo et al. conducted a cross-sectional study in Brazil to find any association between HIV and MS in ART-naive adults. They suggest that HIV increases the risk of MS regardless of ART, and impaired plasma glucose is significantly associated with the presence of MS (p < 0.05) [8]. 

A cross-sectional study in Cameroon by Mbunkah et al. compared the prevalence of MS among ART-exposed, ART-naive, and HIV(-) control patients. MS was significantly more common in ART-exposed patients, 26.5% of the patients with MS had hyperglycemia. MS was significantly frequent in the ART-exposed group, especially in patients receiving first-line drugs (p = 0.022). The prevalence of MS was highest in patients on lamivudine/stavudine/nevirapine regimen (50%) [12]. 

Kingery et al. conducted a study in Tanzania, and they demonstrated that ART was associated with an increased risk of MS. MS was significantly more common in the ART-exposed group (11.3%) compared to the HIV-negative control group (3.3%) (p = 0.04). In this study, 11 of the 17 patients with MS had high fasting blood glucose [13]. 

A study in 2017 by Muhammad et al. compared the rates of MS between ART-naive and ART-exposed patients in Nigeria. The prevalence of MS in patients receiving ART was 19.3% compared to 5.3% in ART-naive patients. Insulin resistance identified by homeostatic model assessment for insulin resistance (HOMA-IR) was prevalent in 79.3% of the ART-exposed patients compared to the 25.0% ART-naive group (p = 0.008). A particular class of ART or more extended periods of ART-exposure was not significantly associated with insulin resistance [14].

Maganga et al. also concluded that glucose metabolism disorders were significantly more prevalent among ART-exposed patients (32.7%) compared to ART-naive (8.0%) and control groups (7.2%) (p < 0.001). DM and impaired glucose tolerance were also more common in patients receiving ART (p = 0.001 and p < 001, respectively). Longer duration of ART or exposure to PIs were not significantly associated with glucose metabolism disorders [18]. 

A cross-sectional study by Levitt et al. compared the dysglycemia prevalence in a South African patient group [20]. According to their findings, dysglycemia was more common in the patients receiving second-line ART (37.0%) compared to patients on first-line ART (26.0%), ART-naive patients (21.6%) and community-based survey (18.0%). The risk of dysglycemia was significantly increased in HIV(+) patients (p < 0.001) [20].

Araujo et al. conducted a study in 2017 to compare the prevalence of insulin resistance among different ART regimens and found that insulin resistance is less prevalent in patients using new antiretroviral regimens compared to old therapies. The prevalence of insulin resistance was 21%, significantly higher in patients on PIs (28%) compared to patients on NNRTIs (14%) (p < 0.01) [23].

According to Jain et al., HCV was significantly associated with the development of DM in HIV-infected patients (p < 0.01). In this study, initiation of PIs was not significantly associated with the occurrence of DM [29].

Dyslipidemia

Three types of lipoproteins are associated with atherosclerotic cardiovascular disease (ASCVD). Low-density lipoprotein (LDL) cholesterol is the most atherogenic form in the blood. Very low-density lipoprotein (VLDL) is another atherogenic lipoprotein, while HDL is not known as an atherogenic type of cholesterol. Atherogenicity of chylomicron is not fully known. According to American Heart Association guidelines, LDL levels of <100 mg/dL and total cholesterol levels of <150 are ideal. The same guideline also suggests that a 1% decrease in LDL levels results in a 1% decrease in ASCVD risk. Table 3 contains the studies we have included about dyslipidemia [43].

**Table 3:** Description of selected studies about dyslipidemia from the review NRTI, nucleotide reverse transcriptase inhibitor; NNRTI, non-nucleoside reverse transcriptase inhibitor; PI, protease inhibitor; ART, antiretroviral therapy; DM, diabetes mellitus; MS, metabolic syndrome; HDL, high-density lipoprotein

Author	Drug studied	Number of patients	Type of study	Result	Conclusion
Bune et al. (2019) [19]	NRTI + NNRTI + PI	633	Cross-sectional study	Prevalence of MS was slightly higher in ART-exposed group compared to ART-naive. Dyslipidemia was present in 60.4% of the ART-exposed compared to 56.9% in ART-naive.	Frequency of MS was slightly higher in ART-exposed patients.
Osoti et al. (2018) [6]	Tenofovir/lamivudine or zidovudine/ lamivudine + nevirapine or efavirenz.	300	Cross-sectional study	Low HDL levels were more common in ART-naive compared to ART-exposed group.	Traditional risk factors weigh more than ART for the development of MS in HIV(+) patients.
Calza et al. (2017) [16]	ART	586	Cross-sectional study	MS was more prevalent in ART-exposed patients than ART-naive. Most common components for MS were high triglyceridemia and low HDL. PI-exposure increased the risk for MS while integrase inhibitor-exposure decreased the risk.	Risk for MS development was higher in ART-exposed group.
Anyabolu et al. (2017) [10]	-	393	Cross-sectional study	Prevalences for high LDL, low HDL, hypertriglyceridemia and elevated total cholesterol were 17.6%, 34.4%, 9.9%, 11.4%, respectively, in ART-naive patients.	Dyslipidemias were common in the patient group.
Fontas et al. (2004) [24]	Single PI, dual PI or NNRTI	7483	Prospective cohort study	Triglyceride and LDL levels were lowest in ART-naive group. Those levels were especially higher in dual-PI group. NNRTI-exposed group had similar triglyceride levels to ART-naive. Low HDL was least frequent in NNRTI-exposed. Risk of low HDL was high in patients receiving single or dual PI.	NNRTIs were better in this study compared to PIs with respect to dyslipidemias.

In some studies, ART-naive individuals were at risk of having dyslipidemia before the ART-initiation. Osoti et al. found that traditional factors were a more significant predictor for MS than ART in a study group with 164 ART-exposed and 136 ART-naive individuals. According to this study, MS was slightly more prevalent in patients receiving ART (16.9%) than ART-naive (15.2%), but this finding was not statistically significant (p > 0.05). Almost all patients were receiving NRTI (99%), while 92% and 6.7% were on NNRTI and PI, respectively. 56% of the patients were receiving nevirapine-based triple therapy (tenofovir/lamivudine/nevirapine) while efavirenz-based triple therapy (tenofovir/lamivudine/efavirenz) was the second most common regimen with a prevalence of 36%. Prevalence of high triglyceride levels was similar in both ART-exposed and ART-naive patients, but low HDL levels were significantly more common in ART-naive patients (p = 0.003) [6].

Anyabolu et al. concluded a cross-sectional study in South-East Nigeria with 393 patients to demonstrate the HIV effect on dyslipidemia in 2017. In their study, the prevalence of different types of dyslipidemias was 17.6% for high serum LDL, 11.4% for elevated total cholesterol, 9.9% for high serum triglycerides, and 34.4% for low serum HDL. Dyslipidemia and CD4 lymphocyte count were significantly associated (p = 0.027) [10].

Calza et al. also suggested that MS risk increased in ART-exposed patients. In this cross-sectional study, MS was significantly higher in the ART-exposed group; high triglycerides and low HDL were the most common features of MS and levels of total cholesterol, triglycerides, and LDL was significantly higher while HDL levels were significantly lower in patients receiving ART. NNRTIs rilpivirine and efavirenz were not significantly associated with the development of MS while dual-PI therapies darunavir/ritonavir (OR: 1.89, p = 0.014) and atazanavir/ritonavir (OR:1.61, p = 0.039) had a significant positive association with MS. The decreased risk of having MS in integrase inhibitor receiving patients was not statistically significant [16].

Bune et al. conducted a cross-sectional study in Southern Ethiopia involving 422 ART-exposed and 211 ART-naive patients to compare the prevalence of MS in ART-exposed and ART-naive patients. MS was prevalent in 22.0% in the patients receiving ART while 20.9% in the ART-naive patients; the most common component of MS was dyslipidemia. Dyslipidemia was seen in 59.2% of the patients, and the prevalence was 60.4% and 56.9% in ART-exposed and ART-naive, respectively; high triglyceride levels (37%) and low HDL levels (34.3%) were the most frequent types of dyslipidemia [19].

Fontas et al. conducted a prospective cohort study with 7483 patients in 2004. In the study, triglyceride, total cholesterol, and LDL levels were lowest in the ART-naive group and highest in patients receiving two different PIs. Patients receiving NNRTIs had close triglyceride levels to ART-naive patients. There was no significant difference among groups for HDL levels, but it was highest in the group receiving NNRTI. In terms of risk of dyslipidemia, PI-exposed patients had a significantly higher risk of high triglycerides and total cholesterol/HDL levels. High total cholesterol risk was significantly higher in the double-PI group compared to the ART-naive group. Each ART-exposed groups were compared to each other about the risk of dyslipidemia as well; patients on double-PI therapy were at a significantly higher risk of dyslipidemias barring LDL and HDL levels. On the other hand, the risk of abnormalities in LDL, HDL, triglycerides, and total cholesterol/HDL levels were significantly lower in the NNRTI-exposed group compared to other drug-exposed groups. Within PIs, ritonavir (RTV)-exposed patients had a significantly higher risk of high total cholesterol levels than nelfinavir (NLF), and high total cholesterol/HDL levels than indinavir (IDV); patients receiving saquinavir (SQV) had the lowest risk of high total cholesterol/HDL. Concerning NNRTIs, patients on efavirenz (EFV) had a significantly higher risk of high triglyceride levels than patients on nevirapine (NVP) while both groups were at a similar degree of risk to have high total cholesterol levels [24].

Hypertension

According to WHO, hypertension is one of the most common disorders in the world; 1.13 billion people in the world have hypertension, even more, when prehypertensive patients are added to the equation [44]. Per American Heart Association and American College of Cardiology’s latest guideline of high blood pressure, categories of blood pressure are: normal levels [systolic blood pressure (SBP): ≤120 mmHg and diastolic blood pressure (DBP): ≤80 mmHg], elevated (SBP: 120-129 mmHg, DBP: <80 mmHg), stage 1 hypertension (SBP between 130 and 139 mmHg, DBP between 80 and 89 mmHg), and stage 2 hypertension (SBP ≥140 mmHg and DBP ≥90 mmHg) [45]. High blood pressure may be a result of HIV or ART, according to some studies we collected.

Muhammad et al. conducted a cross-sectional study in northwestern Nigeria to assess the prevalence of MS among 150 ART-naive and 150 ART-exposed patients [14]. In the ART-exposed group, 135 of the patients were receiving first-line therapy, and 15 patients were receiving second-line therapy [12]; the prevalence of MS in the ART-exposed group was 29 (19.3%), while it was 8 (5.3%) in the ART-naive group [14]. High blood pressure was the most common feature of MS in both ART-exposed and ART-naive patients, 82.8% and 87.5%, respectively, but these findings were not statistically significant (p = 0.61) [14]. High blood pressure was considerably associated with the occurrence of MS (p < 0.001) [14].

The effect of ART was evaluated in a cross-sectional study by Dimala et al. in Cameroon, 100 first-line ART-exposed patients, and 100 ART-naive patients were involved in this study. Hypertension was significantly more prevalent in the ART-exposed patients than the ART-naive group; 38% and 19%, respectively (p = 0.003). Average SBP and DBP values were also higher in ART-exposed patients, 131 ± 31 mmHg and 80 ± 13 mmHg and 125 ± 19 mmHg and 77 ± 12 mmHg, respectively, but these findings were not statistically significant (p = 0.06 and p = 0.118) [17].

In a cross-sectional study that included 422 ART-exposed and 211 ART-naive patients by Bune et al., the prevalence of MS in ART-exposed was 22.5% while it was 20.9% in ART-naive. The prevalence of hypertension in ART-naive was 57.3% compared to 56.9% in ART-exposed patients, and hypertension was present in about 92% of the patients with MS. Hypertension was the only feature of MS that was more prevalent in ART-naive than ART-exposed [19].

Limitations

We could not find enough information about the impact of different drug classes on hypertension, some studies compare the effect of classes on dyslipidemia and insulin resistance, but similar studies are not available for hypertension. Another limitation of our review is most studies we included are cross-sectional studies; thus, there is not much information about the causality.

## Conclusions

The effect of HIV or ART on MS is not entirely understood yet. Multiple factors probably play a role in the occurrence of those metabolic alterations. In our review, we came to the following conclusions: both HIV and ART may have a role in the development of metabolic disorders; these adverse effects are more common in women, people in high BMI, and older people. Also that PIs are the most dangerous class of drugs concerning metabolic disorders. There are not many studies that explain the pathophysiology of HIV/ART-related metabolic alterations; further information about their mechanism is mandatory to prevent or treat these conditions. A detailed comparison of frequencies of ART-related metabolic alterations among different races is also another valuable information we do not have. Further research about genes or proteins in various races that affect the rate or severity of MS in patients on ART can be conducted after this review. A study that enlightens the reasons for the higher prevalence of metabolic alterations in women can be undertaken as well.
